# Supplementary material for: Two-dimensional bilayer ice in coexistence with three-dimensional ice without confinement
Source: Nat Commun. 2024 Jul 9;15:5762. doi: 10.1038/s41467-024-50187-2 (PMC11233582; doi:10.1038/s41467-024-50187-2)
Supplement: Supplementary file 1 — Supplementary Information [file 41467_2024_50187_MOESM1_ESM.pdf]

SUPPLEMENTARY INFORMATION

Two-dimensional bilayer ice in coexistence with three-dimensional ice without confinement

Jing Jiang[1], Yuanming Lai[1, 2]*, Daichao Sheng[3], Guihua Tang[4], Mingyi Zhang[1], Dong Niu[5], Fan Yu[1]

[1] State Key Laboratory of Frozen Soil Engineering, Northwest Institute of Eco-Environment and Resources, CAS, Lanzhou 730000, PR China; [2] Institute of Future Civil Technology, Chongqing Jiaotong University, Chongqing 400074, PR China; [3] School of Civil and Environmental Engineering, University of Technology Sydney, Ultimo, NSW 2007, Australia; [4] MOE Key Laboratory of Thermo-Fluid Science and Engineering, School of Energy and Power Engineering, Xi'an Jiaotong University, Xi'an 710049, PR China; [5] Naval Architecture and Ocean Engineering College, Dalian Maritime University, Dalian 116026, PR China. ✉email: ymlai@lzb.ac.cn

Supplementary Figures

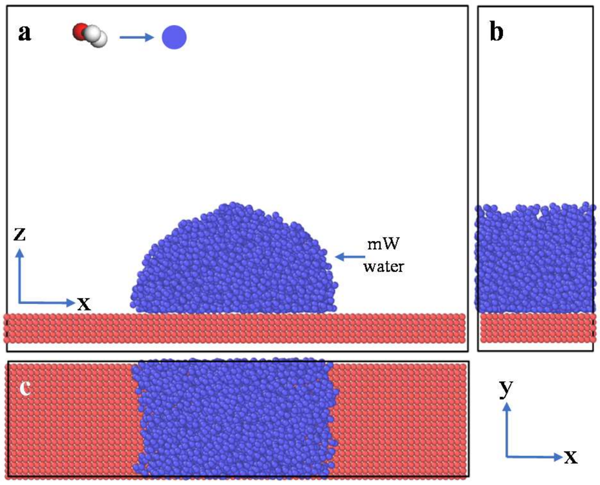

Supplementary Fig. 1 Liquid-solid simulation system. a Front view. **b** Left view. **c** Top view. The studied system contains 2430 water molecules and 4000 Pt atoms. The Pt atoms with lattice constant of 3.92 Å are arranged in face-centered cubic (FCC). The *x* and *y* directions are both periodic boundary conditions, *z* direction is non-periodic boundary conditions, and reflection boundary condition is set at the top. The simulated box size is 15.69 nm × 3.92 nm × 11.77 nm.

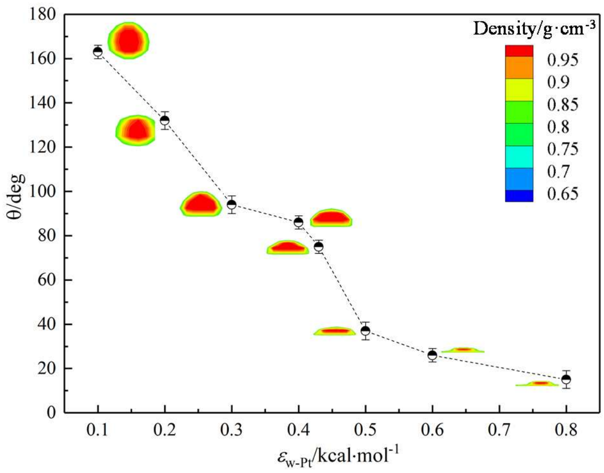

Supplementary Fig. 2 Relationship between contact angle *θ* and surface energy parameter *ε*. The range of energy parameter $\varepsilon_{w\text{-}Pt}$ is 0.1-0.8 kcal·mol^{-1}, covering the range of solid surface from superhydrophobic to superhydrophilic. The contact angles of the surfaces with different wettabilities are analyzed and calculated by the average density profile method. After the relaxation at 300 K reaches the equilibrium state, the droplet density cloud map is obtained by density distribution time averaging calculation, and the droplet boundary contour line is further obtained by density boundary criterion to calculate the apparent contact angle of droplets. A density colorbar shows the meaning of the contact angle heatmaps. As the energy parameter *ε* increases, the contact angle decreases. Data are presented as mean values +/- SD, *n*=5.

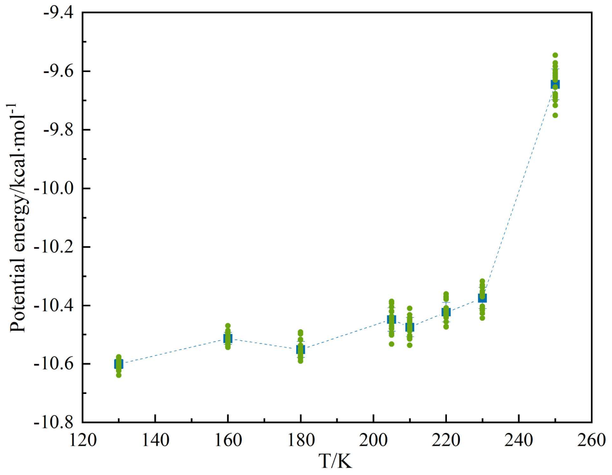

Supplementary Fig. 3 Potential energy per water molecule vs temperature *T*. Variation of the potential energy per water molecule on the solid surface with temperature for energy parameter of 0.5 kcal·mol^{-1}. Data are presented as mean values +/- SD, *n*=20. As the temperature increases, the potential energy per water molecule basically increases. The potential energy per water molecule at 250 K is significantly higher than that of per water molecule at other temperatures.

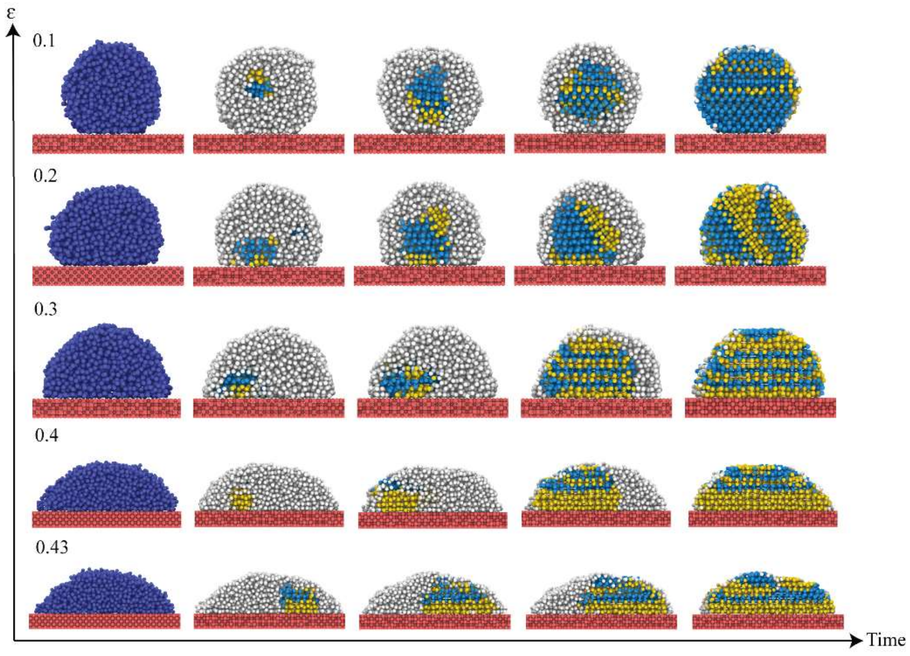

Supplementary Fig. 4 3D disordered ice buildup dynamic formation process. The dynamic processes of nanodroplets on surfaces with different energy parameters (0.10-0.43 kcal·mol^{-1}) show characteristics of the nanodroplets supercooled to nucleation and crystallization. The nucleation process is a phase-transition activation process. Overcoming the free energy barrier to form critical nucleus, the nucleus grows spontaneously. Dark yellow and light blue balls represent hexagonal ice and cubic ice, respectively.

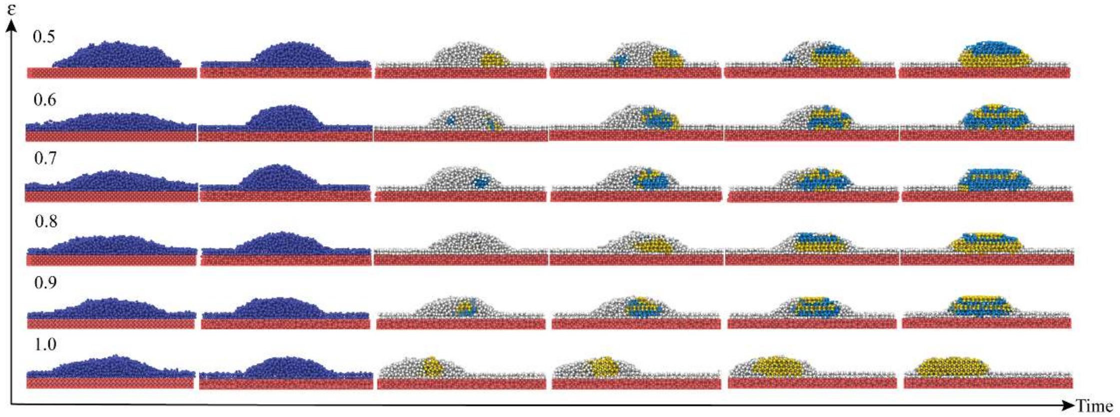

Supplementary Fig. 5 2D-3D ice dynamic formation process on surfaces. It illustrates the characteristics of the nanodroplets supercooled to nucleation and crystallization with different energy parameters (0.5-1.0 kcal·mol^{-1}). The nanodroplets first form 2D ice and then form 3D ice during the supercooled process. Overcoming the free energy barrier to form critical nucleus, the 3D ice nucleus grows spontaneously. Dark yellow and light blue balls represent hexagonal ice and cubic ice, respectively.

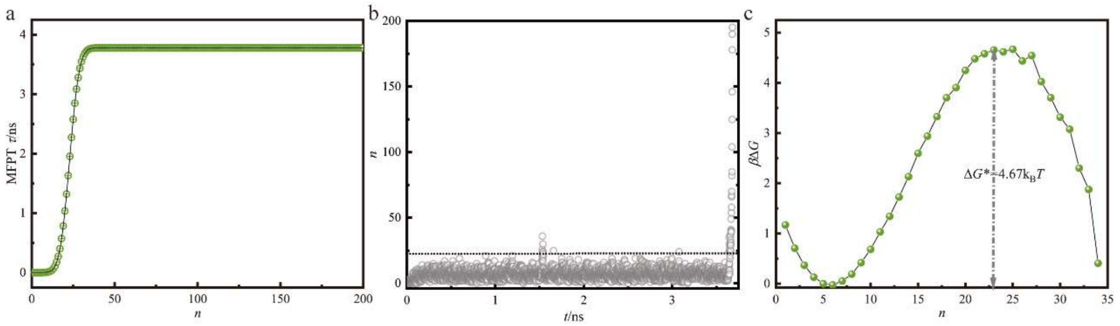

Supplementary Fig. 6 The free energy for ice nucleation process at *T*=205K (*ε*=0.4kcal·mol^{-1}). The free energy barrier is calculated using the kinetic reconstruction method [1] during the ice nucleation process. This method requires two quantities: the steady-state probability distribution $P_{st}(n)$, which represents the probability of finding the system in a state with the largest ice nucleus having n ice molecules, and the mean first-passage time (MFPT), $\tau(n)$, which is defined as the average time needed for the largest nucleus to contain n molecules for the first time [2]. **a** Mean first-passage time (MFPT) $\tau(n)$ as a function of the cluster size n obtained from the MD simulations. To obtain these quantities, more than 60 independent simulations with different initial conditions were performed by MD simulations. For each simulation, the size of the largest cluster in the system is continuously monitored at regular intervals (every 1000 time steps), and the corresponding first appearance time for each size n, $t_i(n)$, is recorded. The mean first-passage time $\tau(n)$ for each size n is simply obtained by averaging $t_i(n)$ over multiple repetitions of simulation with different initial

configurations. Additionally, we obtain the steady-state probability $P_{st}(n)$: at every 1000 time steps during each repetition, we sample the size n of the largest cluster in the system. Subsequently, a histogram is constructed by counting how frequently a given size n occurs as the largest cluster. By normalizing this count with respect to the total number of sampled cluster sizes, we derive the steady-state probability distribution $P_{st}(n)$. The MFPT curve has a sigmoidal shape that reaches a well-defined plateau at larger values of n, indicating that overcoming critical size is rate-limiting step in cluster formation and subsequent growth of cluster takes negligible time compared to activation time. **b** Growth curves of the largest cluster from the MD simulations. **c** Kinetic reconstruction of the free energy of cluster formation obtained from the MD simulations (β=1/k_BT). It depicts $\beta\Delta G$ as a function of the number of ice molecules n in the largest nucleus for this case. It can be observed that $\beta\Delta G$ increases with increasing nucleus size after the inception of a small ice nucleus. When the nucleus reaches its critical size, n=23, $\beta\Delta G$ approaches its maximum value. Subsequently, as the ice nucleus continues to grow, the free energy decreases and triggers further ice formation. The value ΔG^*=4.67k_BT represents the energy difference between the maximum free energy and the minimum free energy after inception of an ice nucleus. ΔG^*=4.67k_BT is associated with the development of the ice nucleus on the solid wall.

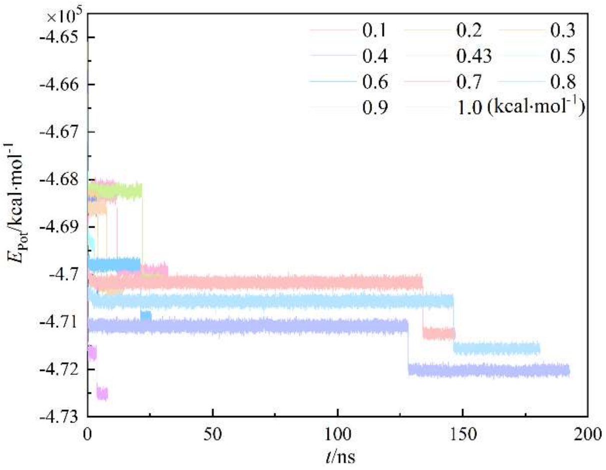

Supplementary Fig. 7 Variation of the potential energy E_{pot}. The range of energy parameter ε is 0.1-1.0 kcal·mol^{-1}, covering the range of solid surface from superhydrophobic to superhydrophilic. The potential energy changes with time during the nucleation process of nanodroplets after quenching and cooling. The sudden drops in E_{pot} mean the occurrence of nucleation event.

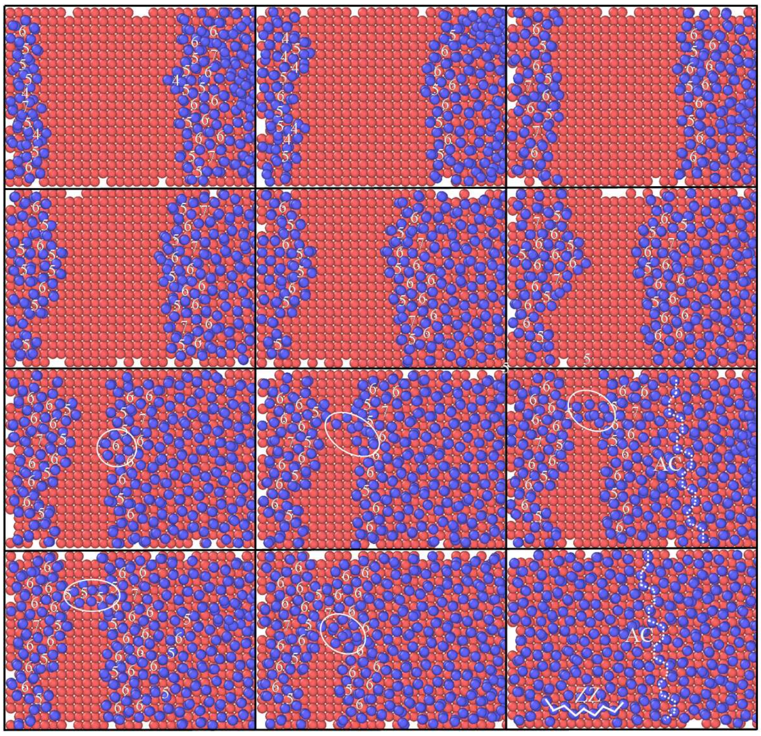

Supplementary Fig. 8 2D ice edge merging growth dynamics. Firstly, the edges of the 2D ice on both sides are formed by the non-rotating stacking of double-layer 4-, 5-, 6-, 7-membered ring ice. Then, with the diffusion of water molecules, the 2D ice growth edge is composed of double-layer 5-, 6-, 7-membered ring water molecules. At the moment of merging at the edge of the 2D ice double layer, the 6-membered ring in the white circle in the picture contributes a single water molecule, which is connected with the left 5-membered ring, and then forms two metastable 4-membered rings to connect the water molecules on both sides. After that, the two 4-membered ring water molecules become relatively stable 5-membered ring water molecules, and the water molecules on both sides merge and grow. During the growth process, the zigzag growth mode and the armchair growth mode of the 2D ice appears.

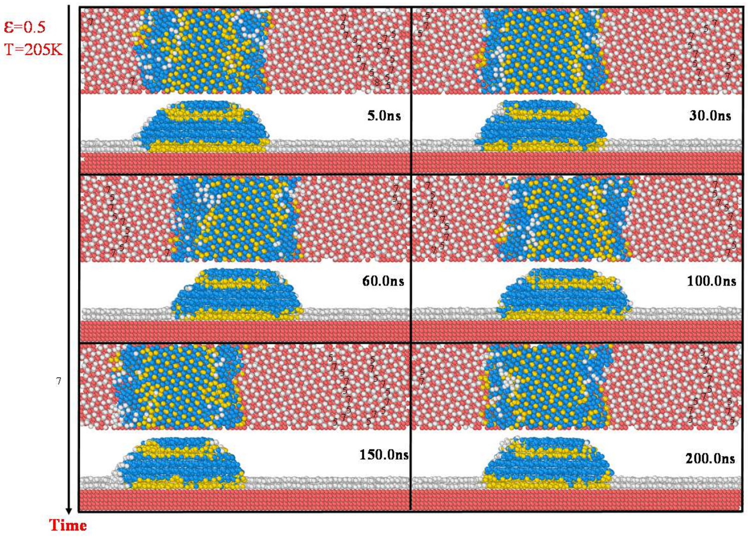

Supplementary Fig. 9 Top view and front view of 2D-3D ice structure variation. In the NVT ensemble, after 200ns of calculation, the 2D-3D coexisting ice structures are relatively stable. The 2D ice is still composed of double-layer 5-, 6-, 7-membered ring water molecules, and the proportion of 5-, 7-membered ring water molecules is basically unchanged, indicating that the 2D-3D coexisting ice is not sensitive to the calculation time when it is sufficiently long.

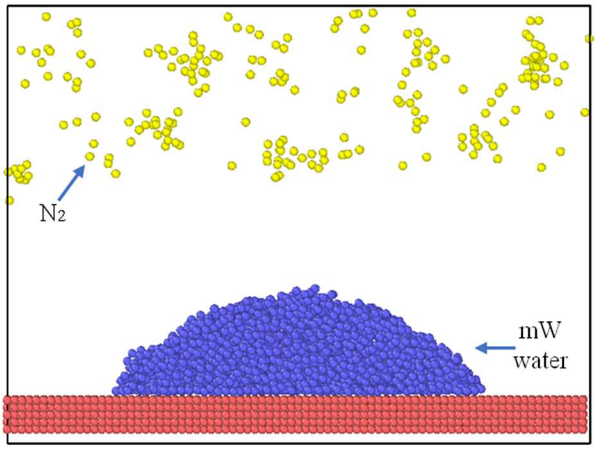

Supplementary Fig. 10 Nitrogen controlled pressure simulation system. Nitrogen molecules adopt the coarse-grained model. The studied system contains 2430 water molecules and 4000 Pt atoms. The number of nitrogen molecules is set to 20, 50, 100, 200 and 300 representing approximately 1.0, 2.5, 5.5, 11.0 and 17.5 atmospheres, respectively. Light yellow balls represent nitrogen molecules.

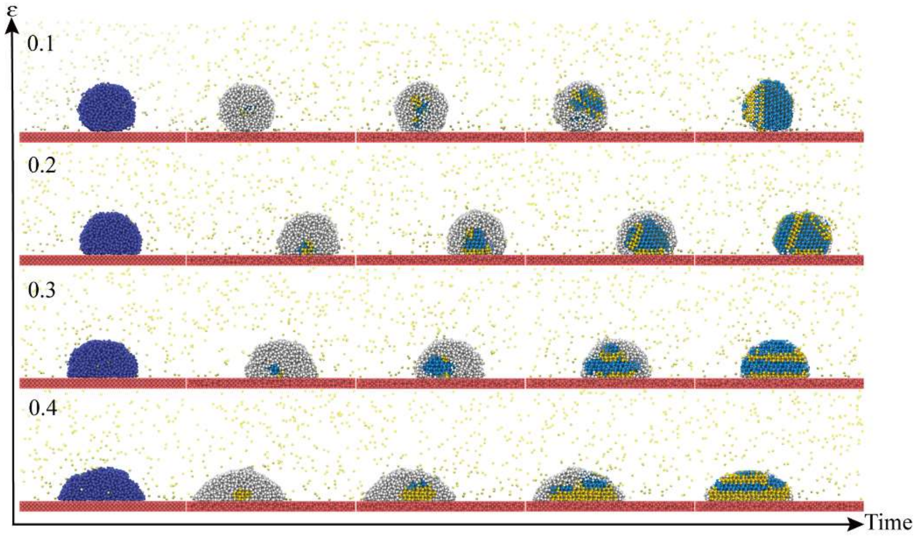

Supplementary Fig. 11 3D disordered ice buildup dynamic formation process at *T*=205K. The number of nitrogen molecules is 200, representing that the system pressure is about 11.0 atm. For ε=0.1-0.4 kcal·mol^{-1}, 3D ice is formed after droplet quenching. For ε=1.0 kcal·mol^{-1}, the formation of ice nuclei occurs inside the droplet, which belongs to homogeneous nucleation. For ε=0.2-0.4 kcal·mol^{-1}, ice nucleation belongs to heterogeneous nucleation because nucleation occurs at the position of the droplet near the solid wall.

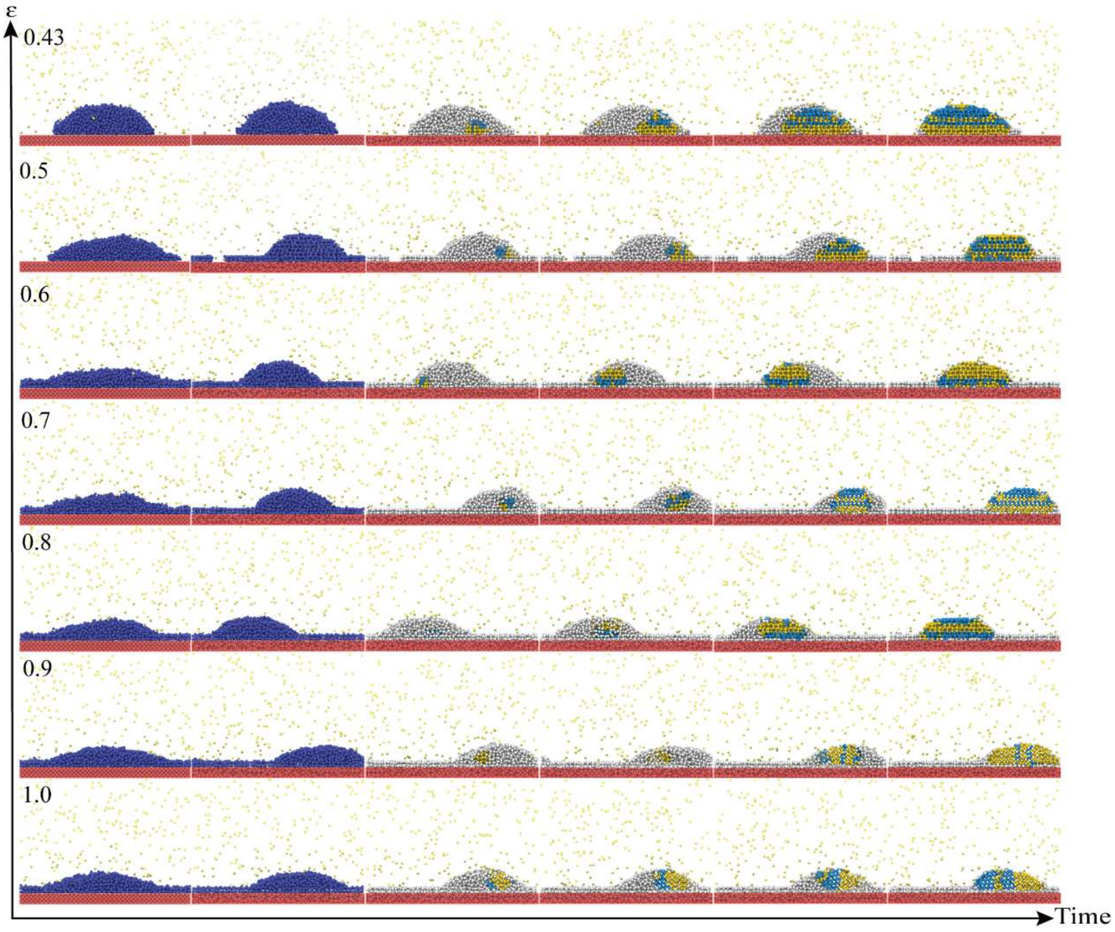

Supplementary Fig. 12 Snapshot of 2D and 3D coexistence ice. For ε=0.43-1.0 kcal·mol^{-1}, 2D and 3D coexisting ice is formed after droplet supercooled. The water molecules near solid surface diffuse to form 2D ice. After overcoming the free energy, the 3D disordered accumulation ice is formed. 2D ice includes two structures, one is composed of double-layer 5-, 6-, 7-membered ring water molecules, and the other is composed of a layer of water molecules with 5,6,7-membered rings and a layer of water molecules with 4,6-membered rings near solid surface.

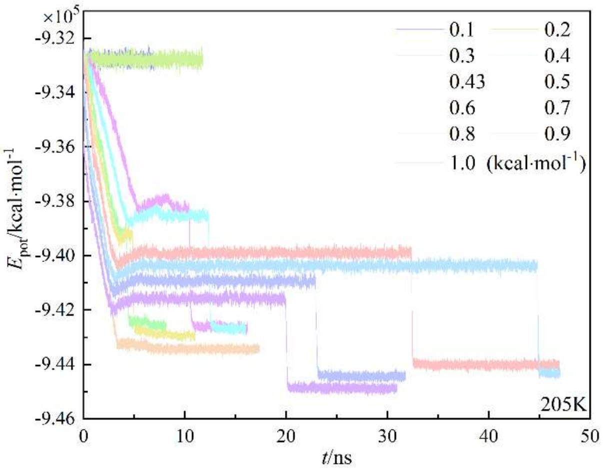

Supplementary Fig. 13 Variation of the potential energy E_{pot} with time t after quenching. A

sudden drop in potential energy means the formation of ice critical nuclei and crystallization.

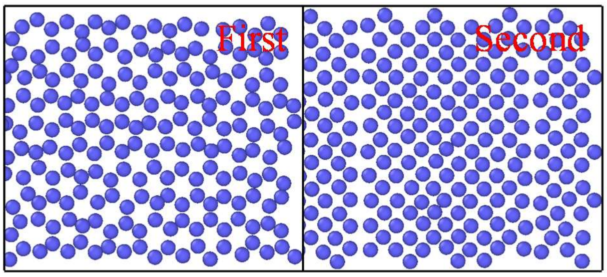

Supplementary Fig. 14 First layer and second layer ice strctures of 2D ice in coexisting ice. For 0.8 kcal·mol^{-1} ≤ ε ≤ 1.0 kcal·mol^{-1}, the first layer ice structure of 2D ice in coexisting ice is composed of 5-, 6-, 7-membered ring water molecules, and the second layer ice structure near solid surface is composed of 4-, 6-membered ring water molecules.

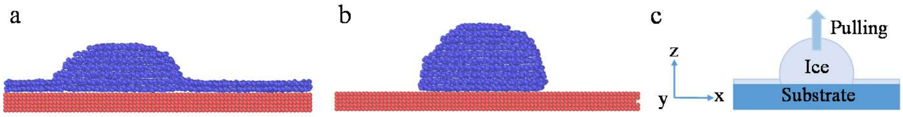

Supplementary Fig. 15 Deicing model in MD simulations. a 2D and 3D coexisting ice model. **b** 3D ice model. **c** Illustration of the detachment process with increasing pulling force acting on ice.

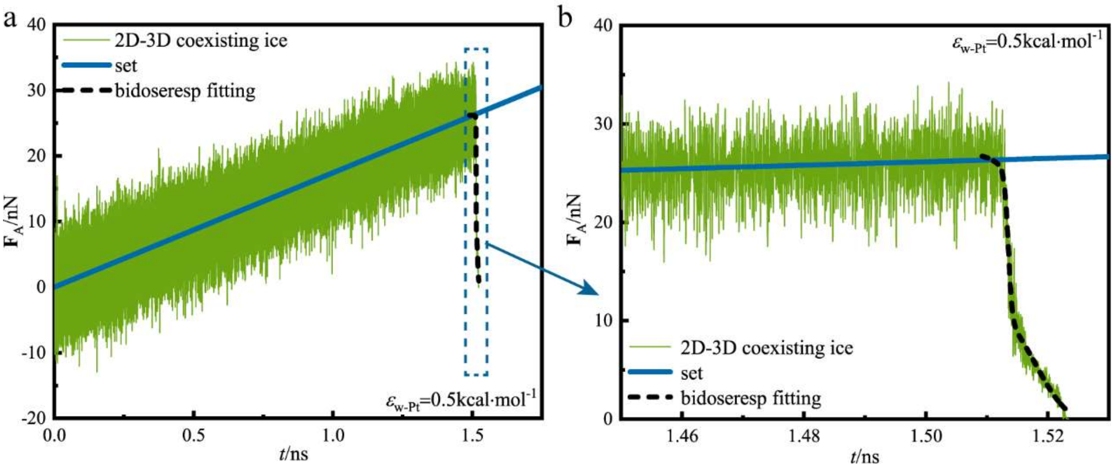

Supplementary Fig. 16 2D and 3D coexisting ice adhesion force calculation procedure. a Ice adhesion force of 2D ice in coexistence with 3D ice on Pt surface with ε=0.5 kcal·mol^{-1}. $\mathbf{F}_A$ is the fluctuating adhesion force between ice and solid substrate. Set is the pulling force acting on ice as a function of time. It represents that the initial value of acceleration is 0 and increases linearly with 5.734*10^{-9} nm • fs^{-2}. Bidoseresp fitting is the least-square fitting of the datas for the part below the "set" line using the bidirectional-dose-response function. **b** The enlarged view of the area surrounded by blue dashed lines. The ice adhesion force was determined by the intersection between the "set" line and the extrapolated biphasic dose-response function.

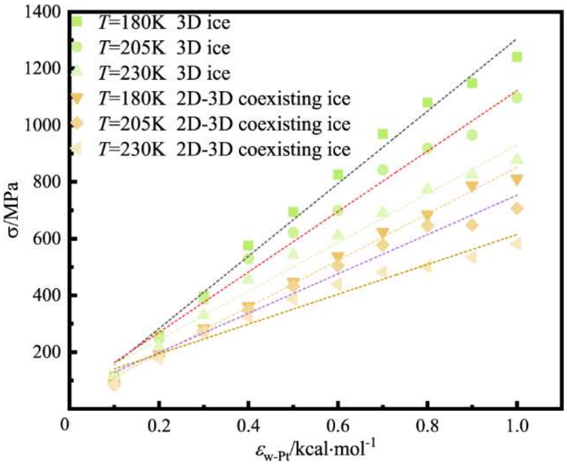

Supplementary Fig. 17 Relationship between the ice adhesion strength σ and wettability energy parameter *ε*. For the 2D-3D coexisting ice and 3D ice systems for different temperature working conditions, the linear correlation slope of $\varepsilon_{\text{w-Pt}}$ of coexisting ice on solid surface is smaller than that of 3-dimensional ice on solid surface. The dash lines are obtained by linear fitting the datas.

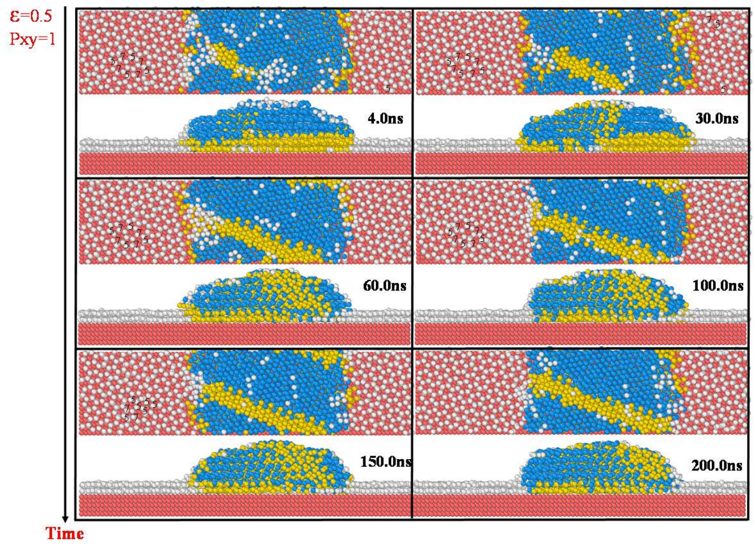

Supplementary Fig. 18 Top view and front view of 2D and 3D coexisting ice structure variation at 205K. In NP$_{xy}$T ensemble, the pressure is set to P_{xy}=1atm. The studied system contains 2430 water molecules and 4000 Pt atoms. The *x*, *y* and *z* directions are all periodic boundary conditions. With the increase of calculation time, the 5-, 7-membered rings in the double-layer ice are reduced. After 200ns of calculation, the 2D ice composed of metastable double-layer 5-, 6-, 7-membered ring water molecules is transformed into steady-state 6-membered ring 2D ice. 3D ice structure is not sensitive to the calculation time.

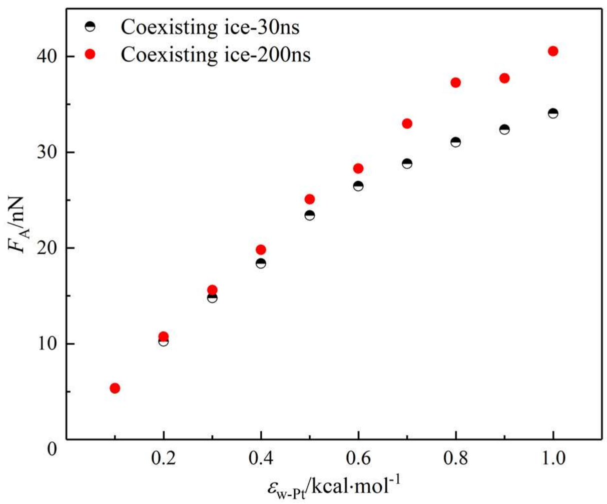

Supplementary Fig. 19 Relationship between the ice adhesion force F_A and wettability energy parameter $\varepsilon_{w\text{-}Pt}$ for the coexisting ice at 205K. At 30ns, the 2D ice is composed of double-layer 5-, 6-, 7-membered ring water molecules. At 200ns, the 2D ice is composed of double-layer 6-membered ring water molecules. With the increase of energy parameter $\varepsilon_{w\text{-}Pt}$, the ice adhesion force at 200ns is gradually larger than that at 30 ns, which is attributed to the combined effect of surface wetting characteristics, 2D ice structure and ice freezing time.

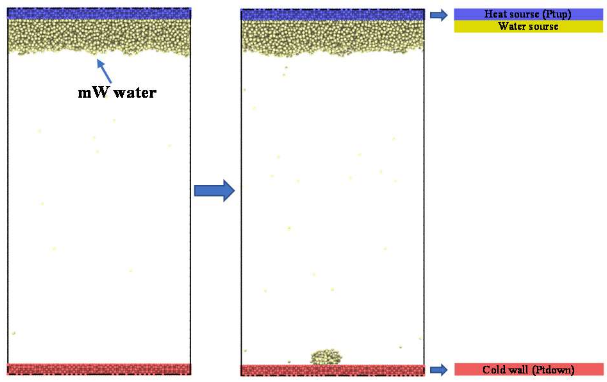

Supplementary Fig. 20 Initial conformation of vapor deposition. The high-temperature surface provides the heat source, and the liquid layer in the high-temperature surface region is the vapor source. As the vapor deposition on the cold surface proceeds, the vapor molecules in the system can be supplemented from the upper liquid layer, thereby maintaining the continuation of the vapor deposition. Red and blue balls represent solid walls. Light yellow balls represent gas water.

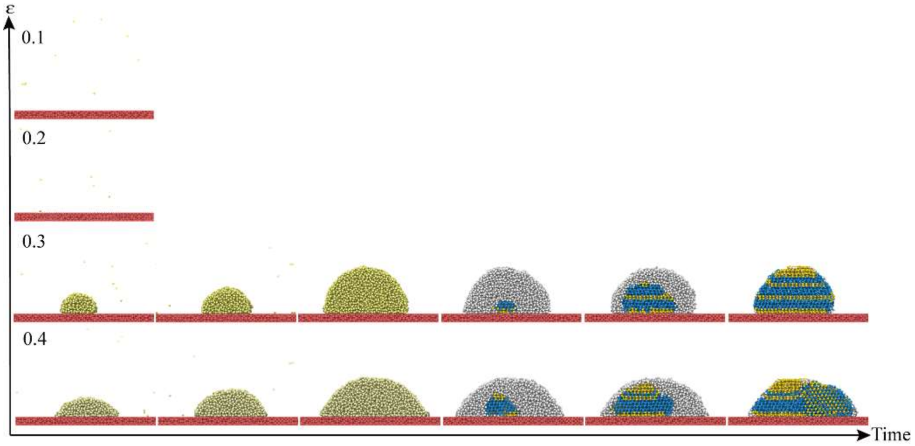

Supplementary Fig. 21 Gas-liquid-3D ice transition. For ε=0.1-0.2 kcal·mol^{-1}, there is only gas phase. For ε=0.3-0.4 kcal·mol^{-1}, with the progress of vapor deposition, the gas-liquid phase variation occurs, and independent nanodroplets appear on the solid surface. The water vapor condenses in dropwise condensation mode, and then the liquid-solid phase transition occurs. With the increase of wettability, the contact angle of the nanodroplet decreases and the contact radius increases, which enhances the interaction between droplet and surface. The nucleation of the nanodroplet preferentially occurs on the solid surface. Dark yellow and light blue balls represent hexagonal ice and cubic ice, respectively.

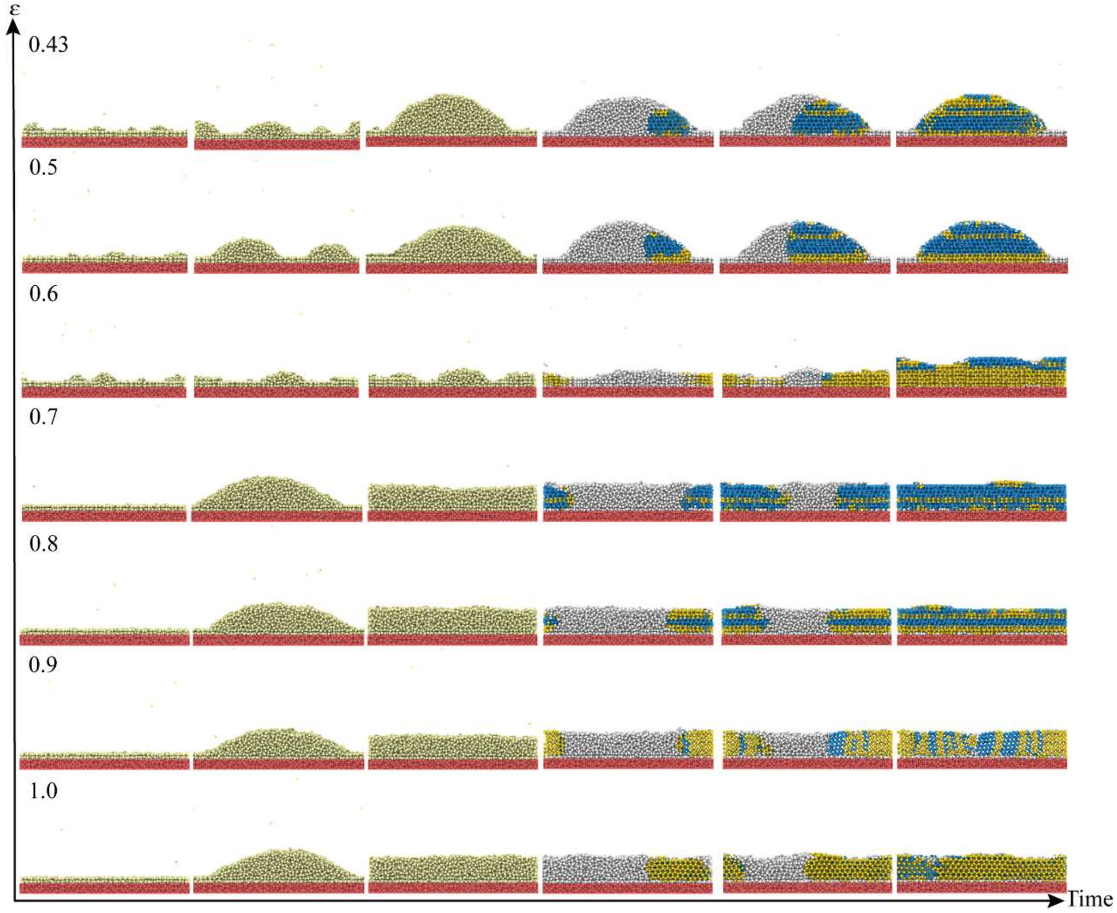

Supplementary Fig. 22 Gas-liquid-2D and 3D coexisting ice/3D ice transition. For ε=0.43-0.5 kcal·mol^{-1}, the gas phase molecules diffuse to form 2D ice. 2D ice promotes the nucleation and growth of 3D ice, thus forming 2D and 3D coexisting ice. For ε=0.6-1.0 kcal·mol^{-1}, overcoming the free energy barrier to form a critical nucleus, the liquid-solid phase transition occurs, the crystal nucleus grows rapidly and crystallizes, finally forming 3D hexagonal structure and cubic structure disordered accumulation ice. Dark yellow and light blue balls represent hexagonal ice and cubic ice, respectively.

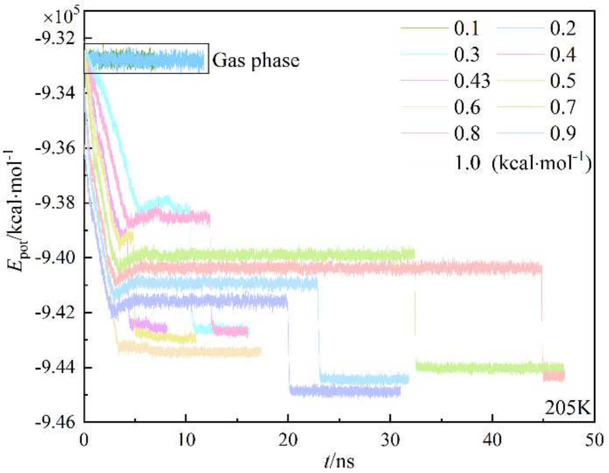

Supplementary Fig. 23 Potential energy E_{pot} variation and surface wettability ε in vapor deposition. The range of energy parameter ε is 0.1-1.0 kcal·mol^{-1}, covering the range of solid surface from superhydrophobic to superhydrophilic. For ε=0.3-1.0 kcal·mol^{-1}, the potential energy of gas molecules drops twice. The first drop is gas-liquid phase transition or the gas-solid phase transition coexists with gas-liquid phase transition, and the second drop is liquid-solid phase transition, which verifies the above different surface condensation and crystallization phase transition characteristics.

References

[1] Wedekind J., Reguera D. Kinetic reconstruction of the free-energy landscape. *J. Phys. Chem. B* 112, 11060-11063 (2008).

[2] Wedekind J., Strey R., Reguera D. New method to analyse simulations of activated processes. *J. Chem.Phys.* 126, 134103 (2007).
